# Aldehyde Dehydrogenase‐2 Alleviates Septic Myocardial Injury by Inhibiting Caspase‐11‐Mediated Noncanonical Pyroptosis

**DOI:** 10.1155/cdr/5289405

**Published:** 2026-01-14

**Authors:** Huan Liang, Yuying He, Yiren Wang, Rui Zhang, Mengjie Yu, Hongwei Ye, JiaHui Wang, Qin Gao

**Affiliations:** ^1^ Key Laboratory of Cardiovascular and Cerebrovascular Diseases, Bengbu Medical University, Bengbu, Anhui, China, bbmc.edu.cn; ^2^ Department of Graduate Management, The First Affiliated Hospital of Bengbu Medical University, Bengbu Medical University, Bengbu, Anhui, China, bbmc.edu.cn; ^3^ Department of Physiology, Bengbu Medical University, Bengbu, Anhui, China, bbmc.edu.cn; ^4^ Department of Anatomy, Bengbu Medical University, Bengbu, Anhui, China, bbmc.edu.cn

**Keywords:** aldehyde dehydrogenase-2, caspase-11, gasdermin-D, noncanonical pyroptosis, sepsis

## Abstract

**Purpose:**

The purpose of this study is to investigate the role of aldehyde dehydrogenase‐2 (ALDH2) in septic myocardial injury, focusing on noncanonical pyroptosis.

**Methods:**

In vivo, C57BL/6J mice were divided into five groups: Sham, cecal ligation and puncture (CLP), CLP + Alda‐1 (ALDH2 agonist), Sham + dimethyl sulfoxide (DMSO, solvent control), and CLP + DMSO. Cardiac function and histological/ultrastructural changes were assessed via echocardiography, hematoxylin–eosin (HE) staining, and transmission electron microscopy (TEM). Tumor necrosis factor‐*α* (TNF‐*α*) and noncanonical pyroptosis‐related proteins (caspase‐11, gasdermin‐D [GSDMD], high mobility group box 1 [HMGB1], and receptor for advanced glycation end products [RAGE]) were measured by enzyme‐linked immunosorbent assay (ELISA) and western blotting. Coimmunoprecipitation (CO‐IP) explored molecular mechanisms. In vitro, H9C2 cells were divided into six groups: Control, lipopolysaccharide (LPS)‐treated, ALDH2‐green fluorescent protein (GFP), LPS + ALDH2‐GFP, GFP, and GFP + LPS. Cell viability, lactate dehydrogenase (LDH) release, creatine kinase isoenzymes (CK‐MB), and target protein levels were detected via spectrophotometry, western blotting, and immunofluorescence (IF).

**Results:**

In vivo, Alda‐1 significantly attenuated CLP‐induced cardiac dysfunction and reduced myocardial histological damage and ultrastructural impairment. In vitro, ALDH2 overexpression lowered LPS‐induced H9C2 cell viability, CK‐MB, and LDH release. Upregulating ALDH2 significantly reduced caspase‐11, HMGB1, and RAGE expression. CO‐IP showed ALDH2 interacted with HMGB1, RAGE, and GSDMD.

**Conclusion:**

ALDH2 protects the myocardium from septic injury by inhibiting caspase‐11‐mediated noncanonical pyroptosis, possibly via direct interactions with GSDMD, HMGB1, and RAGE.

## 1. Introduction

Sepsis is defined as life‐threatening organ dysfunction caused by a dysregulated host response to infection, while septic shock is a subset of sepsis characterized by profound underlying circulatory and cellular/metabolic abnormalities that substantially increase mortality [1]. Sepsis arises from diverse pathogens (notably Gram‐positive and Gram‐negative bacteria) and triggers inflammatory imbalance, immune dysfunction, endoplasmic reticulum stress, mitochondrial damage, and cell death pathways (autophagy, apoptosis, and pyroptosis), ultimately leading to multiple organ dysfunction and complications [2]. Sepsis‐induced cardiomyopathy (SICM), also termed sepsis‐induced myocardial dysfunction (SIMD), is a well‐documented complication of sepsis and septic shock that further causes hemodynamic instability, cardiac dysrhythmias, and cardiac insufficiency [3]. Thus, exploring the molecular mechanisms of SICM and developing novel therapies is urgently required.

Pyroptosis is a type of programmed cell death, mainly classified into canonical and noncanonical subtypes. Accumulating evidence indicates that noncanonical pyroptosis plays a crucial role in sepsis progression [4–7]. Pathogen invasion stimulates pattern recognition receptors (PRRs) on innate immune cells to recognize pathogen‐associated molecular patterns (PAMPs) or danger‐associated molecular patterns (DAMPs) released by damaged cells, inducing inflammatory factor secretion, inflammatory responses, and pyroptosis to resist pathogens. However, inflammatory response imbalance triggers a cytokine storm, leading to massive cell death and irreversible organ damage [2, 5, 8]. Host cell noncanonical pyroptosis is a key mechanism in sepsis, elicited by intracellular lipopolysaccharide (LPS)—the major outer membrane component of Gram‐negative bacteria [9]. As a PAMP, LPS is recognized by toll‐like receptor 4 (TLR4) and initiates intracellular signaling cascades that ultimately activate caspase‐11, a key protein in noncanonical pyroptosis [5, 10]. A previous study [11] reported that extracellular LPS binds directly to high mobility group box 1 (HMGB1); the resulting HMGB1‐LPS complex is then internalized into acidic lysosomes via the receptor for advanced glycation end products (RAGE). HMGB1 permeabilizes the lysosomal phospholipid bilayer, allowing LPS release into the cytosol. Cytosolic LPS then binds and activates caspase‐11, which cleaves the pyroptosis executor gasdermin‐D (GSDMD). GSDMD, acting as a pore‐forming protein, ultimately induces pyroptosis [11, 12].

Aldehyde dehydrogenase‐2 (ALDH2) is localized in the mitochondrial matrix and is highly expressed in organs with high mitochondrial content and high‐energy metabolism, including the liver, lung, heart, and brain [13]. Despite its well‐recognized role in ethanol metabolism, previous studies [13–15] have shown that ALDH2 plays a critical protective role against myocardial injuries, including septic cardiomyopathy, alcoholic cardiomyopathy, diabetic cardiomyopathy, and myocardial ischemia–reperfusion injury. Therefore, this study is aimed at investigating whether ALDH2 protects the heart against SICM by inhibiting noncanonical pyroptosis and verifying the potential underlying mechanisms.

## 2. Methods

### 2.1. Materials

The following materials were used: Alda‐1 (a specific ALDH2 agonist), dimethyl sulfoxide (DMSO, solvent for Alda‐1), and LPS (Sigma, United States); primary antibodies for western blotting (anti‐GAPDH [Absin, China], anti‐caspase‐11, anti‐GSDMD, anti‐HMGB1, anti‐RAGE, and anti‐ALDH2 [Abcam, United Kingdom]); primary antibodies for immunofluorescence (IF) (anti‐caspase‐11, anti‐GSDMD [Santa Cruz Biotechnology, United States]); antibodies for coimmunoprecipitation (CO‐IP) (anti‐ALDH2 [Santa Cruz Biotechnology, United States], IgG2b [Cell Signaling Technology, United States]); secondary antibodies for western blotting (anti‐rabbit IgG‐horseradish peroxidase [HRP] [Absin, China]); secondary antibodies for IF (anti‐rabbit/mouse/rat IgG‐Alexa Flour [Abcam, United Kingdom]); H9C2 cardiomyocytes (Future Biotechnology, China); ALDH2‐overexpressing adenovirus (Weizhen Biosciences, China); 4 ^′^,6‐diamidino‐2‐phenylindole dihydrochloride (DAPI) and protein A+G magnetic beads (Beyotime, China); enzyme‐linked immunosorbent assay (ELISA), lactate dehydrogenase (LDH) assay kit, and creatine kinase isoenzyme assay kit (CK‐MB) (Jiancheng Bioengineering Institute, China); cell counting kit‐8 (CCK‐8, Biosharp, China).

### 2.2. In Vivo Study

#### 2.2.1. Animals and Treatments

Male specific‐pathogen‐free (SPF) C57BL/6J mice (8–12 weeks old, 18–23 g) were purchased from Skbex Biotechnology Co. Ltd. (License No. SCXK [Yu] 2020‐0005). All animal experimental procedures were conducted in accordance with the guidelines of the Animal Ethics Committee of Bengbu Medical University and were approved under Permit No. 072, 2021.

Mice undergoing cecal ligation and puncture (CLP) were fasted for 12 h and anesthetized with 1%–2% isoflurane. Following anesthesia, the abdominal fur was removed and the skin disinfected with an iodophor. A midline abdominal incision (≤ 2 cm) was made along the ventral white line to incise the skin, subcutaneous adipose tissue, abdominal muscle layer, and peritoneum sequentially. The cecum was exteriorized from the abdominal cavity; its distal 1/3 was ligated with 3‐0 sterile silk suture, punctured twice using an 18‐gauge needle, and gently squeezed to extrude a small amount of cecal contents. The cecum was then returned to the abdominal cavity, and the abdominal muscle layer and skin were closed with 4‐0 sterile silk suture. Each mouse′s surgical procedure was completed within 15 min [15, 16].

The mice were randomly divided into five groups (*n* = 6/group): Sham, CLP, CLP + Alda‐1 (ALDH2 agonist), Sham + DMSO (Alda‐1 solvent), and CLP + DMSO groups. The Sham group underwent the same surgical procedure as the CLP group, except that the cecum was neither ligated nor punctured. For mice in the CLP + Alda‐1 group, 10 mg/kg Alda‐1 was administered via intraperitoneal injection 1 h prior to the establishment of the CLP model [17]. As solvent controls, mice in the Sham + DMSO and CLP + DMSO groups received intraperitoneal injections of 10 mg/kg DMSO at corresponding time points: the Sham + DMSO group was injected 1 h before the sham operation, and the CLP + DMSO group was injected 1 h before the CLP model establishment.

#### 2.2.2. Echocardiography

Cardiac function and hemodynamic parameters in each group were evaluated using echocardiography (Visual Sonics, Canada). Mice were anesthetized with 1%–2% isoflurane, and B‐mode and M‐mode images were acquired via the parasternal long‐axis view (PLAX). Left ventricular ejection fraction (LVEF), left ventricular fractional shortening (LVFS), stroke volume (SV), and left ventricular end‐systolic internal diameter (LVESD) were then calculated and analyzed for each mouse using B/M‐mode data.

#### 2.2.3. Hematoxylin–Eosin (HE) Staining

Isolated mouse hearts from each group were fixed in 4% paraformaldehyde, dehydrated in absolute ethanol, and embedded in paraffin for sectioning into 3‐*μ*m thick slices. Sections were stained with HE solution, and myocardial pathological changes were observed using a light microscope (Olympus, Japan).

#### 2.2.4. Transmission Electron Microscopy (TEM)

For TEM analysis, myocardial tissue sections (70 nm) were prepared from mouse hearts in each group as follows: tissue samples were first fixed in 2.5% glutaraldehyde followed by osmic acid, dehydrated in a graded series of absolute ethanol, and embedded in epoxy resin. After ultrathin sectioning, sections were stained with a saturated uranyl acetate solution and lead citrate solution sequentially and then visualized using a TEM (Hitachi, Japan).

#### 2.2.5. ELISAs

Briefly, mouse heart tissue was homogenized and centrifuged, and the resulting supernatant was collected. Subsequent steps for each supernatant were performed according to the ELISA kit instructions. The optical density (OD) at 450 nm was measured using a microplate reader (Bio‐Tek, United States), and the data were analyzed to calculate TNF‐*α* concentrations.

#### 2.2.6. Western Blotting

Total protein was extracted from myocardial tissue using radioimmunoprecipitation assay (RIPA) lysis buffer, and protein concentration was determined with a bicinchoninic acid (BCA) protein assay kit. Protein samples (30 g) were separated by 10% sodium dodecyl sulfate‐polyacrylamide gel electrophoresis (SDS‐PAGE) and then transferred onto polyvinylidene difluoride (PVDF) membranes. Membranes were blocked with 5% nonfat milk in Tris‐buffered saline with Tween‐20 (TBST) for 2 h at room temperature, followed by incubation with primary antibodies overnight (16–20 h) at 4°C: anti‐GAPDH (1:20,000), anti‐caspase‐11 (1:1000), anti‐GSDMD (1:1000), anti‐HMGB1 (1:20,000), anti‐RAGE (1:1000), and anti‐ALDH2 (1:5000). After washing with TBST, membranes were incubated with goat anti‐rabbit secondary antibody conjugated to HRP (1:10,000) for 2 h at room temperature. Finally, protein bands were visualized using an enhanced chemiluminescence (ECL) imaging system (FLI Imaging Systems, United States).

#### 2.2.7. CO‐IP

All CO‐IP experiments were performed according to the manufacturer′s kit instructions. Briefly, protein A+G magnetic beads were first incubated with anti‐ALDH2 antibody at 4°C for 12–16 h to form antibody‐bead complexes. The beads were then washed with phosphate‐buffered saline (PBS) to remove unbound antibody, followed by incubation with myocardial total protein extracts at 4°C for another 12–16 h to allow antigen‐antibody binding. After incubation, the samples were denatured at 95°C for 5 min, and the magnetic beads were discarded by centrifugation. The interacting proteins of ALDH2 (caspase‐11, GSDMD, HMGB1, and RAGE) were detected by western blotting.

### 2.3. In Vitro Study

#### 2.3.1. Cell Culture and Treatment

The H9C2 cardiomyocytes were cultured in high‐glucose Dulbecco′s modified Eagle′s medium (DMEM) supplemented with 10% fetal bovine serum (FBS) and 1% penicillin–streptomycin mixture, under a humidified atmosphere of 5% carbon dioxide (CO_2_) at 37°C.

The H9C2 cardiomyocytes overexpressing ALDH2 were generated via adenovirus transfection at a multiplicity of infection (MOI) of 70, following the protocol provided by Weizhen Biosciences. After transfection, cells were treated with LPS (300 *μ*g/mL) for 24 h and assigned to six groups: Control group, LPS‐treated group (LPS), ALDH2‐overexpression green fluorescent protein (GFP) group, ALDH2‐GFP + LPS group, GFP group, and GFP + LPS group.

#### 2.3.2. Detection of CK‐MB Assay Kit

The H9C2 cells were cultured in 96‐well plates, and cell culture supernatants from each group were collected, with all experimental procedures performed in accordance with the instructions of the CK‐MB assay kit. The absorbance was measured at a wavelength of 340 nm to calculate the final CK‐MB activity.

#### 2.3.3. CCK‐8 Measurement

The H9C2 cardiomyocytes were seeded in 96‐well plates for culture. Following the experimental intervention, CCK‐8 solution was added in accordance with the manufacturer′s instructions. After the recommended incubation period, the OD at 450 nm for each well was measured using a microplate reader to assess cell viability (Bio‐Tek, United States).

#### 2.3.4. LDH Assay

The cell culture supernatants were collected from each group, and LDH activity was measured using 96‐well plates. All samples were processed in accordance with the LDH assay kit′s principles and instructions, and the OD at 450 nm for each well was detected using a microplate reader to calculate LDH levels (Bio‐Tek, United States).

#### 2.3.5. Western Blotting

The cells in each group were first subjected to the respective experimental interventions, and the remaining operations were consistent with those for in vivo protein detection.

#### 2.3.6. IF Measurement

The H9C2 cells were fixed with 4% paraformaldehyde at room temperature for 15–20 min, washed 3 times with PBS (5 min each), and then permeabilized and blocked with 5% BSA + 0.3% Triton X‐100 at 37°C for 1–2 h. After that, cells were incubated with primary antibodies (Caspase‐11 = 1 : 50, GSDMD = 1 : 50, HMGB1 = 1 : 250, RAGE = 1 : 100) at 4°C for 8–12 h and then washed 3 times with PBS. Alexa Fluor–conjugated secondary antibodies (anti‐rabbit/rat/mouse IgG, 1:200) were incubated at 37°C for 30 min in the dark. Nuclei were counterstained with DAPI (5 *μ*g/mL) at 37°C for 6–10 min. Fluorescent signals were visualized using a motorized inverted fluorescence microscope (Zeiss, Germany).

### 2.4. Statistical Analysis

All experimental data were analyzed using GraphPad Prism 6.0 (GraphPad Software, United States). Measurement data were expressed as mean ± standard deviation (mean ± SD). For comparisons among multiple groups, statistical significance was determined by one‐way analysis of variance (ANOVA) followed by Tukey′s multiple comparison test. A value of *p* < 0.05 was considered statistically significant, with significance levels labeled as *p* < 0.05 and *p* < 0.01 in figures to clarify the magnitude of differences.

## 3. Results

### 3.1. In Vivo Study

#### 3.1.1. ALDH2 Protects Cardiac Function Against CLP‐Induced Septic Cardiomyopathy

To investigate the role of ALDH2 in CLP‐induced cardiac function impairment in mice, key indicators of left ventricular systolic function, including LVEF, LVFS, SV, and LVESD, were measured via echocardiography before euthanasia. M‐mode echocardiographic images (Figure 1a) showed that myocardial contractility was impaired in the CLP group, while ALDH2 activation (via Alda‐1) alleviated this impairment. Quantitatively, compared with the Sham group, the CLP group exhibited significantly decreased LVEF, LVFS, and SV and increased LVESD. In contrast, the CLP + Alda‐1 group showed significant improvements compared with the CLP group: LVEF, LVFS, and SV were increased, and LVESD was decreased (Figure 1b). Additionally, myocardial TNF‐*α* levels were markedly elevated in the CLP group and were reduced by Alda‐1 treatment (Figure 1c). These results collectively indicate that ALDH2 activation protects against CLP‐induced cardiac functional injury and inflammation in septic mice.

Figure 1Alda‐1 protects cardiac function from sepsis‐induced injury. (a) M‐mode echocardiography images of each group. (b) Changes in LVEF, LVFS, SV, and LVESD among different groups. (c) Alterations in myocardial TNF‐*α* levels in each group. (Data are presented as mean ± SD, *n* = 5.) ∗∗ indicates *p* < 0.01 versus Sham group; # indicates *p* < 0.05 versus CLP group; ## indicates *p* < 0.01 versus CLP group.

**Figure:**
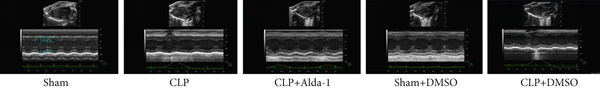
(a)

**Figure:**
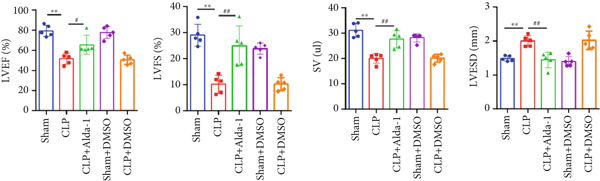
(b)

**Figure:**
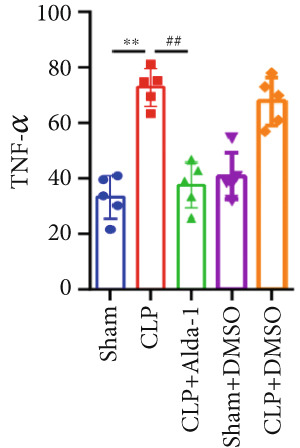
(c)

#### 3.1.2. ALDH2 Ameliorates Myocardial Histopathological and Ultrastructural Injury

To evaluate myocardial structural damage, we performed HE staining and TEM on myocardial tissues from each group (Figure 2).

Figure 2Alda‐1 protects cardiac tissue and ultrastructure from sepsis‐induced injury. (a) HE staining results of myocardial tissue in each group, with red arrows indicating inflammatory cells (magnification: 200×, 400×). (b) Changes in the ultrastructure of myocardial tissue under electron microscopy, with red arrows indicating mitochondria (magnification: 8000×, 15,000×).

**Figure:**
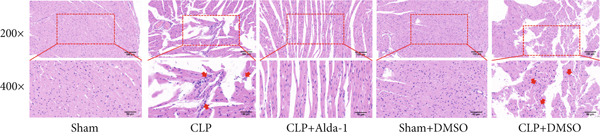
(a)

**Figure:**
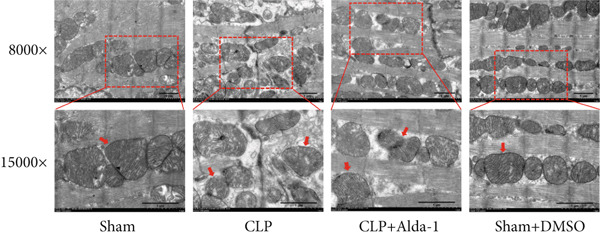
(b)

For HE staining (Figure 2a), the Sham group showed neatly arranged myocardial fibers without obvious inflammatory cell infiltration; the CLP group exhibited severe myocardial tissue necrosis, fiber rupture, and extensive infiltration of inflammatory cells (predominantly neutrophils and macrophages, indicated by red arrows) in the interstitium; after Alda‐1 intervention (CLP + Alda‐1 group), myocardial fibers showed marked repair, and inflammatory cell infiltration was significantly reduced; in the Sham + DMSO and CLP + DMSO groups, the results showed no significant differences when contrasted with the Sham and CLP groups, respectively, confirming no interference effect of DMSO.

For TEM observation (Figure 2b), the Sham group had morphologically intact mitochondria with clear cristae and regularly arranged sarcomeres; the CLP group displayed obvious mitochondrial swelling, vacuolation, cristae dissolution, disordered sarcomere arrangement, and irregular cytoplasmic vesicles/organelle debris (red arrows), consistent with pyroptosis ultrastructural characteristics; in the CLP + Alda‐1 group, mitochondrial damage was alleviated and sarcomere arrangement became more regular; the Sham + DMSO and CLP + DMSO groups showed no notable deviations from their corresponding Control groups.

These findings collectively indicate that ALDH2 activation via Alda‐1 mitigates both histopathological and ultrastructural myocardial injury induced by CLP.

#### 3.1.3. ALDH2 Downregulates the Expression of Noncanonical Pyroptosis‐Related Proteins (Caspase‐11, GSDMD, HMGB1, and RAGE) in CLP Mice

For the purpose of investigating ALDH2′s regulatory role in noncanonical pyroptosis‐related proteins, we measured the myocardial expression levels of caspase‐11, GSDMD, HMGB1, RAGE, and ALDH2 via western blotting (Figure 3). Compared with the Sham group, myocardial ALDH2 expression was significantly increased in the CLP + Alda‐1 group (Figure 3a,d), confirming effective ALDH2 activation by Alda‐1. Meanwhile, the CLP group exhibited significantly elevated protein levels of caspase‐11, GSDMD, HMGB1, and RAGE compared with the Sham group (Figures 3b, 3c, 3e, 3f, 3g, and 3h). Notably, Alda‐1 treatment (CLP + Alda‐1 group) significantly suppressed the upregulation of these noncanonical pyroptosis markers: caspase‐11, GSDMD, HMGB1, and RAGE compared with the CLP group.

Figure 3Alda‐1 reduces the expression of proteins related to noncanonical pyroptosis. (a–c) Representative blots of ALDH2, caspase‐11, GSDMD, HMGB1, and RAGE. (d–h) Statistical analysis of the relative expression levels of each protein. (Data are presented as mean ± SD, *n* = 5.) ∗ indicates *p* < 0.05 versus Sham group; ∗∗ indicates *p* < 0.01 versus Sham group; # indicates *p* < 0.05 versus CLP group; ## indicates *p* < 0.01 versus CLP group; && indicates *p* < 0.01 versus CLP + Alda‐1 group.

**Figure:**
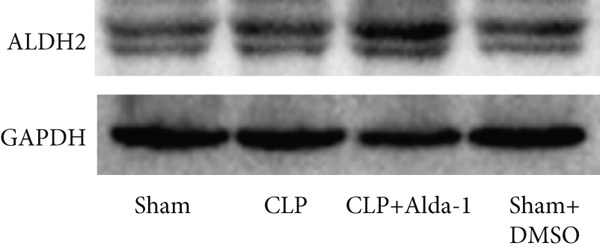
(a)

**Figure:**
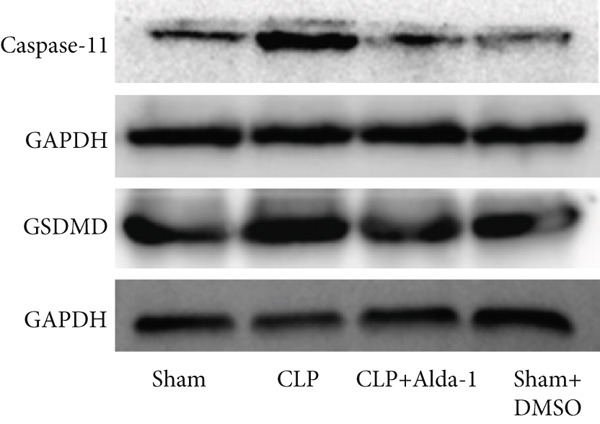
(b)

**Figure:**
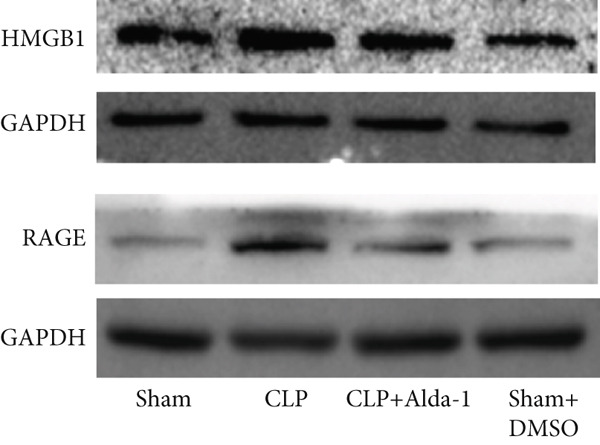
(c)

**Figure:**
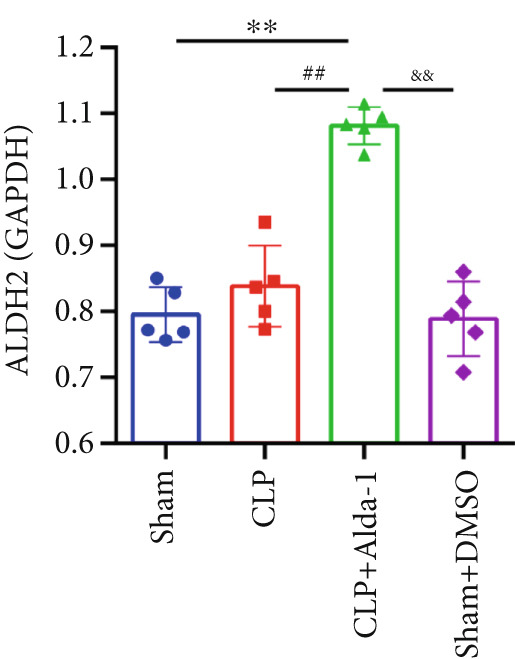
(d)

**Figure:**
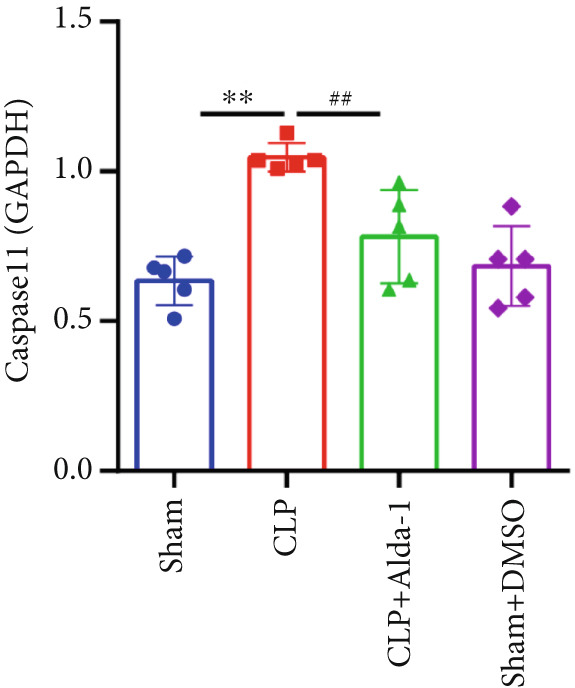
(e)

**Figure:**
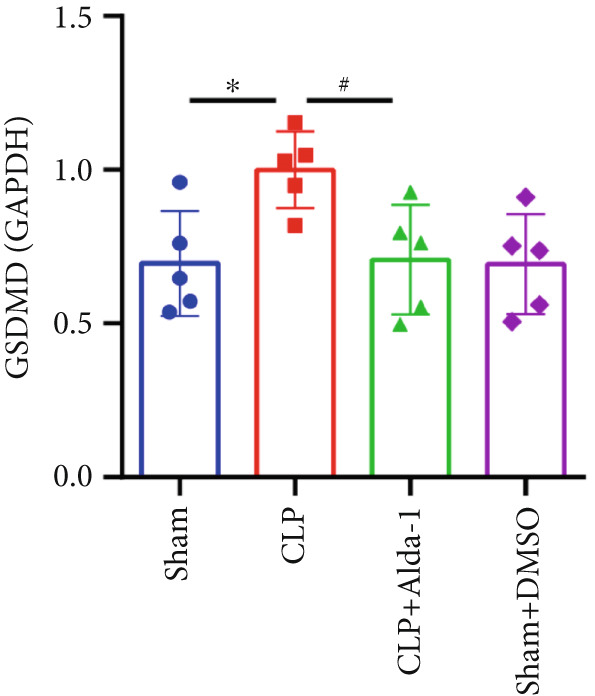
(f)

**Figure:**
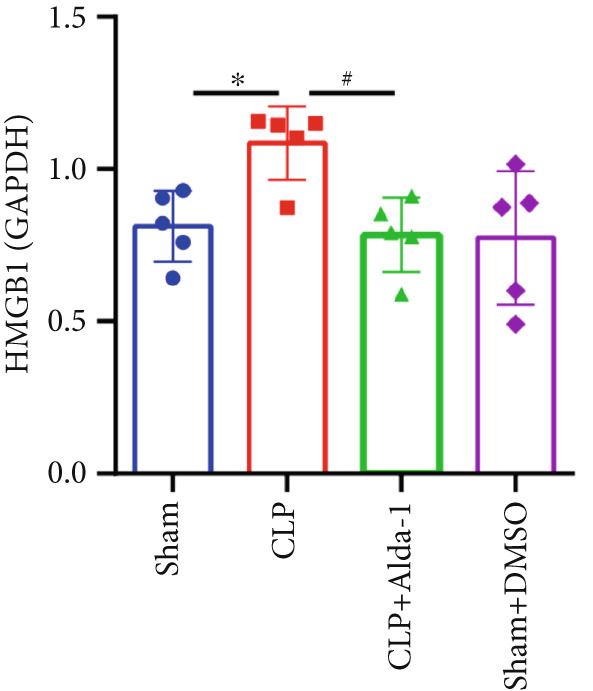
(g)

**Figure:**
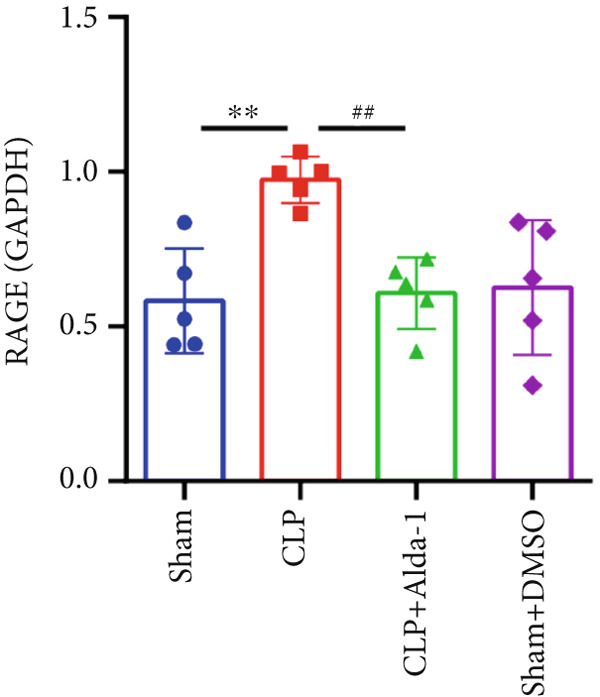
(h)

These findings indicate that Alda‐1 enhances ALDH2 expression and inhibits myocardial noncanonical pyroptosis in CLP‐induced septic mice by downregulating caspase‐11, GSDMD, HMGB1, and RAGE.

#### 3.1.4. ALDH2 and HMGB1/RAGE/GSDMD Interact Strongly to Form a Protein Complex

To elucidate the mechanistic basis of ALDH2 in septic myocardial protection, we analyzed protein–protein interactions (PPIs) using the STRING database and CO‐IP experiments (Figure 4).

Figure 4The mechanism by which ALDH2 protects the myocardium from sepsis‐induced injury may be related to GSDMD, HMGB1, and RAGE. (a) Panoramic network of PPI during pyroptosis. (b) Core protein interaction network of noncanonical pyroptosis. Circles represent proteins, with node color and structure indicating different proteins; lines (edges) represent protein–protein interactions, with thicker and darker lines indicating stronger supporting evidence for the interaction. (c) Gene Ontology enrichment. FDR: false discovery rate (corrected significance level), with lighter colors indicating stronger significance (smaller *p* value). Gene count: the size of the circle represents the number of genes involved in the process. (d) Blots showing ALDH2 interaction with GSDMD, caspase‐11, HMGB1, and RAGE in Sham and CLP groups. (e) Statistical analysis of interaction intensity between ALDH2 and target proteins in Sham and CLP groups. The *y*‐axis represents the normalized interaction intensity (IP/input grayscale ratio). Blue bars denote the Sham group, and red bars denote the CLP group. (Data are presented as mean ± SD, *n* = 5.) ∗ indicates *p* < 0.05 versus Sham group; ∗∗ indicates *p* < 0.01 versus Sham group.

**Figure:**
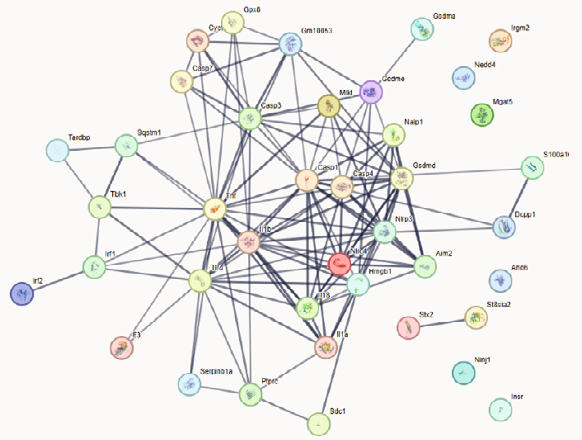
(a)

**Figure:**
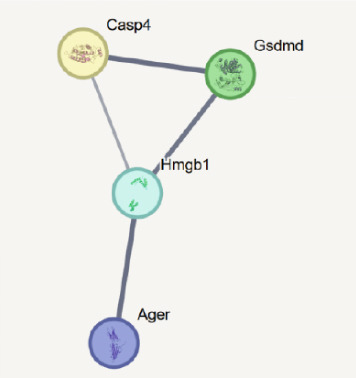
(b)

**Figure:**
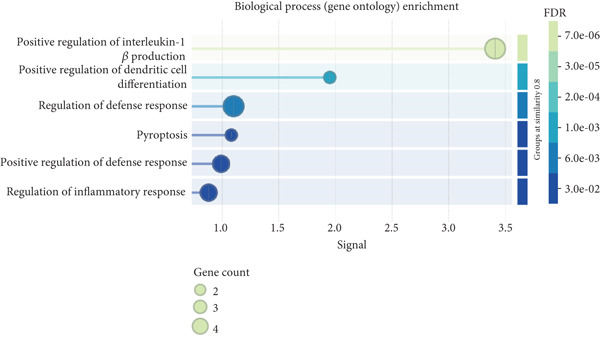
(c)

**Figure:**
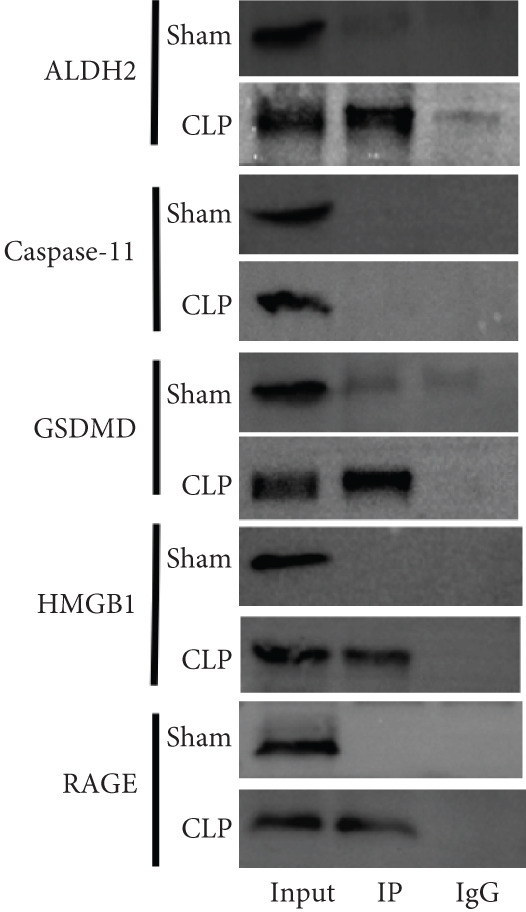
(d)

**Figure:**
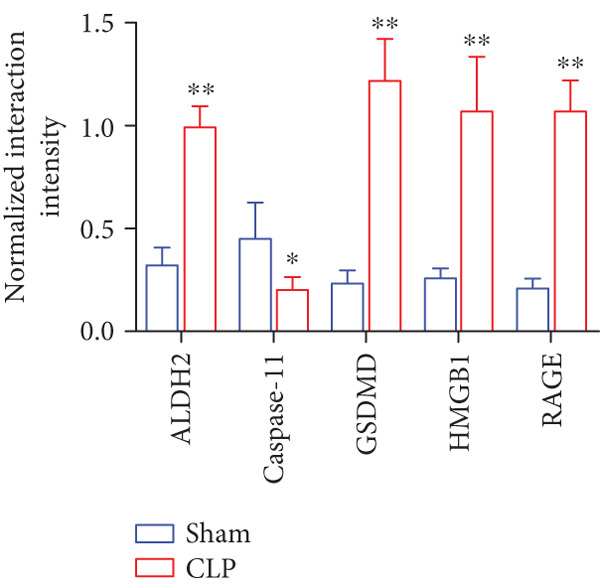
(e)

Via the STRING database, the panoramic protein interaction network during pyroptosis (Figure 4a) revealed a dense regulatory network involving inflammasome components and pyroptosis executors, implying complex synergistic or antagonistic relationships among these proteins in inflammation activation and cell death processes. Focusing on the noncanonical pyroptosis pathway, core proteins including caspase‐4 (a key molecule of the human noncanonical inflammasome, functionally analogous to murine caspase‐11), GSDMD, HMGB1, and RAGE were identified, with their direct interaction network (Figure 4b) further clarifying the core regulatory nodes of noncanonical pyroptosis. Functional enrichment analysis (Figure 4c) confirmed significant enrichment of these factors in pyroptosis processes, consistent with their high interaction propensity under septic pathological conditions.

Then, via CO‐IP experiments assessing protein interactions in the Sham and CLP groups (Figure 4d,e), in the Sham group, interaction bands of ALDH2 with HMGB1, RAGE, and GSDMD were barely detectable, while in the CLP group, these interaction bands were markedly enhanced. Statistical analysis of the “grayscale value ratio of target protein in IP fraction to that in Input fraction” showed that the normalized interaction intensity of ALDH2 with HMGB1, RAGE, and GSDMD was significantly higher in the CLP group than in the Sham group (Figure 4e).

These findings indicate that ALDH2 interacts specifically with HMGB1, RAGE, and GSDMD under CLP‐induced septic conditions, forming a protein complex. This “ALDH2‐HMGB1/RAGE/GSDMD” axis likely mediates the protective effect of ALDH2 against septic myocardial injury, providing a key mechanistic clue for further investigation.

### 3.2. In Vitro Study

#### 3.2.1. Changes in CK‐MB, LDH, and Cell Viability Across Groups

With the aim of assessing LPS‐induced myocardial cell injury and the protective effect of ALDH2, we measured CK‐MB, LDH, and cell viability in H9C2 cells. For CK‐MB levels (Figure 5a), compared with the Control group, CK‐MB levels were significantly elevated in the LPS group; overexpression of ALDH2 (ALDH2‐GFP + LPS group) significantly reduced CK‐MB levels compared with the LPS group. For cell viability (Figure 5b), the LPS group showed significantly decreased cell viability compared with the Control group, while ALDH2 overexpression significantly increased cell viability compared with the LPS group. For LDH levels (Figure 5c), LDH levels in the LPS group were significantly higher than those in the Control group; in the ALDH2‐GFP + LPS group, LDH levels were markedly decreased compared with the LPS group. There were no significant differences in CK‐MB levels, LDH levels, and cell viability between the Control group and the Control + ALDH2‐GFP group as well as the Control + GFP group, indicating that adenovirus exerted no interfering effect. Collectively, these results indicate that ALDH2 overexpression alleviates LPS‐induced H9C2 cell injury.

Figure 5Overexpression of ALDH2 alleviates LPS‐induced myocardial cell injury. (a) Analysis of CK‐MB levels in each group. (b) Analysis of cell viability detected by CCK8 in each group. (c) Analysis of LDH levels in each group. (Data are presented as mean ± SD, *n* = 5.) ∗∗ indicates *p* < 0.01 versus Control group; ## indicates *p* < 0.01 versus LPS group.

**Figure:**
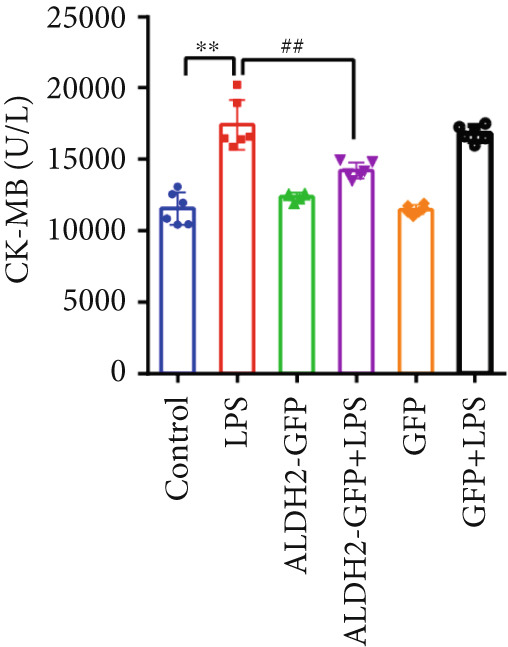
(a)

**Figure:**
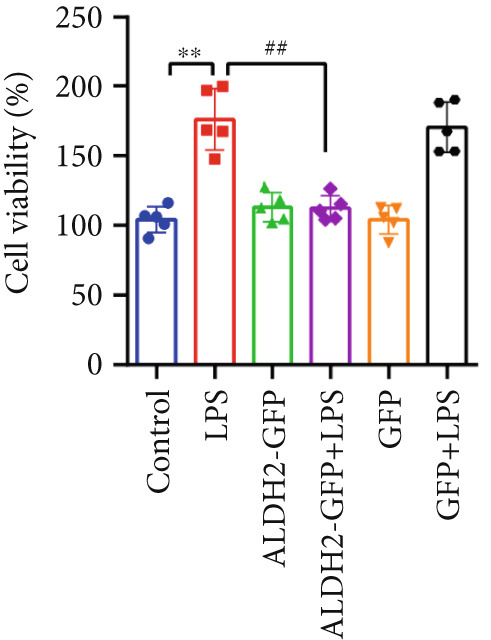
(b)

**Figure:**
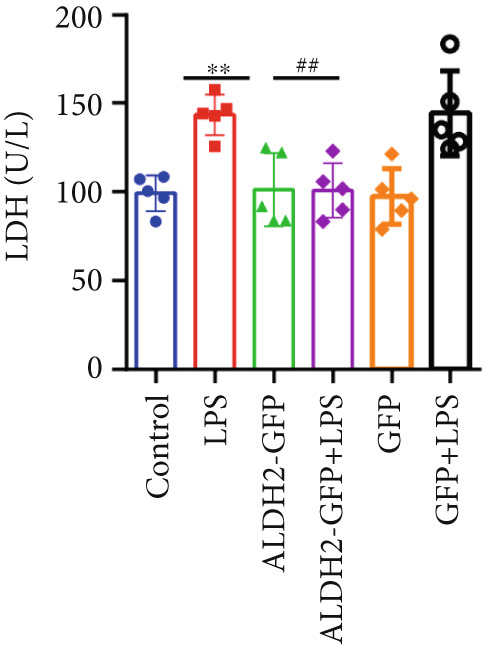
(c)

#### 3.2.2. ALDH2 Suppresses the Expression of Caspase‐11, GSDMD, HMGB1, and RAGE in LPS‐Treated H9C2 Cells

To investigate the effect of ALDH2 on noncanonical pyroptosis‐related proteins in vitro, we measured the expression of ALDH2, caspase‐11, GSDMD, HMGB1, and RAGE in H9C2 cells via western blotting (Figure 6).

Figure 6Overexpression of ALDH2 reduces the expression of proteins related to the noncanonical pyroptosis. (a, b) Representative blots of ALDH2, caspase‐11, GSDMD, HMGB1, and RAGE. (c–g) Statistical analysis of the relative expression levels of each protein. (Data are presented as mean ± SD, *n* = 5.) ∗∗ indicates *p* < 0.01 versus Control group; # indicates *p* < 0.05 versus LPS group; ## indicates *p* < 0.01 versus LPS group; && indicates *p* < 0.01 versus ALDH2‐GFP group.

**Figure:**
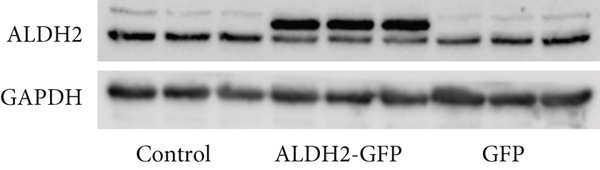
(a)

**Figure:**
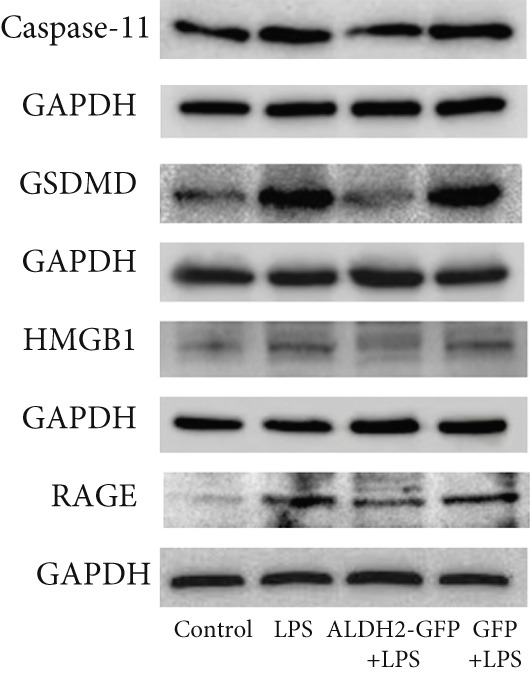
(b)

**Figure:**
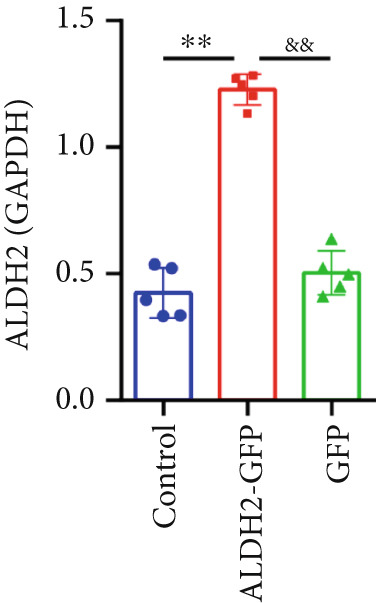
(c)

**Figure:**
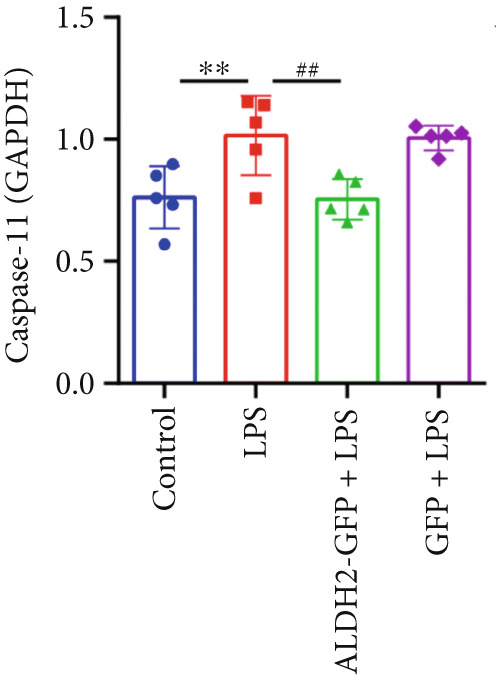
(d)

**Figure:**
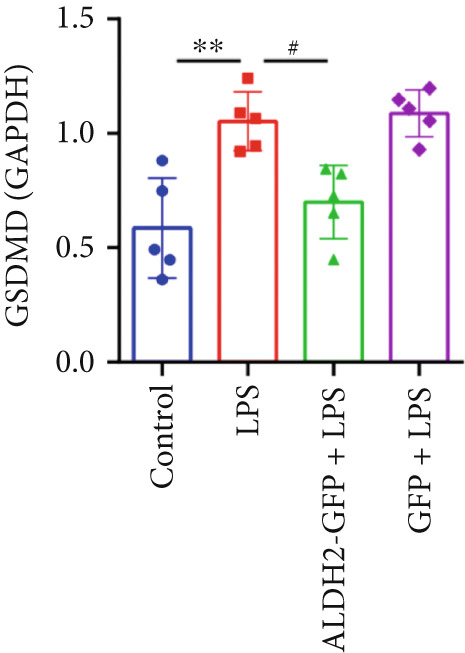
(e)

**Figure:**
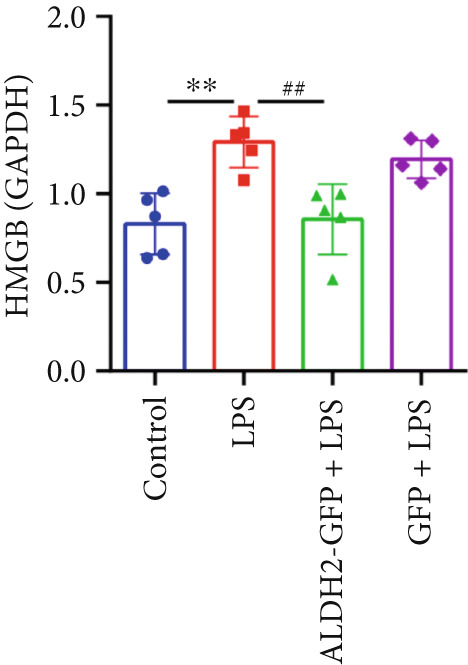
(f)

**Figure:**
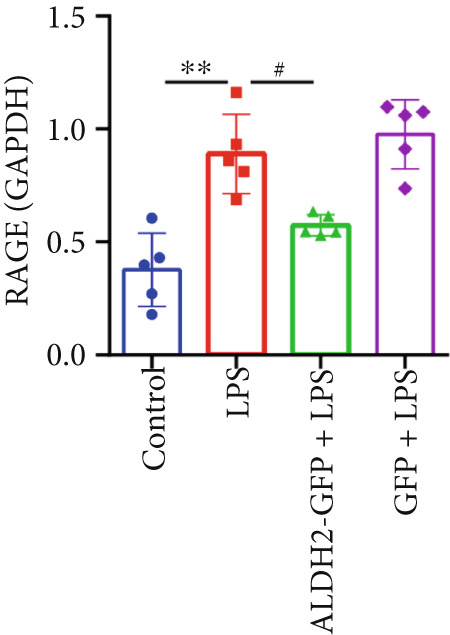
(g)

Western blotting results (Figure 6a,c) showed that ALDH2 expression was significantly increased in the ALDH2‐GFP group, confirming successful overexpression of ALDH2 by adenovirus transfection. Compared with the Control group, the LPS group exhibited significantly elevated protein levels of caspase‐11, GSDMD, HMGB1, and RAGE (Figures 6b, 6d, 6e, 6f, and 6g). Notably, ALDH2 overexpression (ALDH2‐GFP + LPS group) significantly suppressed the upregulation of these noncanonical pyroptosis markers: caspase‐11, GSDMD, HMGB1, and RAGE compared with the LPS group.

These findings indicate that ALDH2 overexpression inhibits the expression of noncanonical pyroptosis‐related proteins (caspase‐11, GSDMD, HMGB1, and RAGE) in LPS‐treated H9C2 cells, suggesting that ALDH2 may exert its protective effect by suppressing noncanonical pyroptosis in cardiomyocytes.

#### 3.2.3. ALDH2 Inhibits Noncanonical Pyroptosis as Evidenced by IF

Consistent with the western blotting results, IF (Figure 7) showed that the red fluorescence intensities of caspase‐11 and GSDMD were significantly increased in the LPS group. In contrast, in the LPS + ALDH2‐GFP group, the fluorescence intensities of caspase‐11 and GSDMD were significantly decreased. These findings indicate that ALDH2 reduces the expression of noncanonical pyroptosis key factors (caspase‐11 and GSDMD) in LPS‐induced H9C2 cells, thereby alleviating cell injury by inhibiting noncanonical pyroptosis.

Figure 7Overexpression of ALDH2 reduces the expression of noncanonical pyroptotic proteins induced by LPS stimulation. (a) IF staining images of caspase‐11 (red), GSDMD (red), and DAPI (blue). (b) Fluorescence intensity analysis of caspase‐11 and GSDMD (200×, scale bar = 100 *μ*m). (Data are presented as mean ± SD, *n* = 5.) ∗ indicates *p* < 0.05 versus Control group; ∗∗ indicates *p* < 0.01 versus Control group; # indicates *p* < 0.05 versus LPS group; ## indicates *p* < 0.01 versus LPS group.

**Figure:**
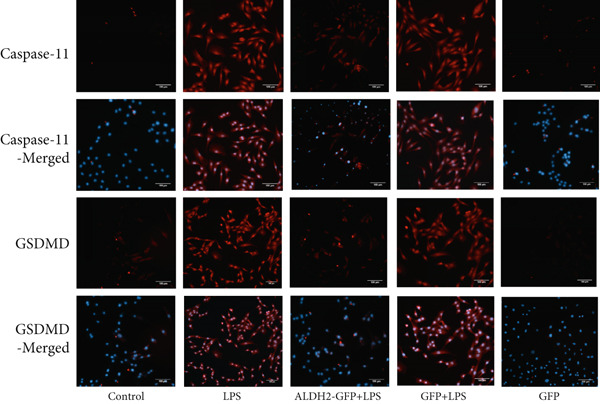
(a)

**Figure:**
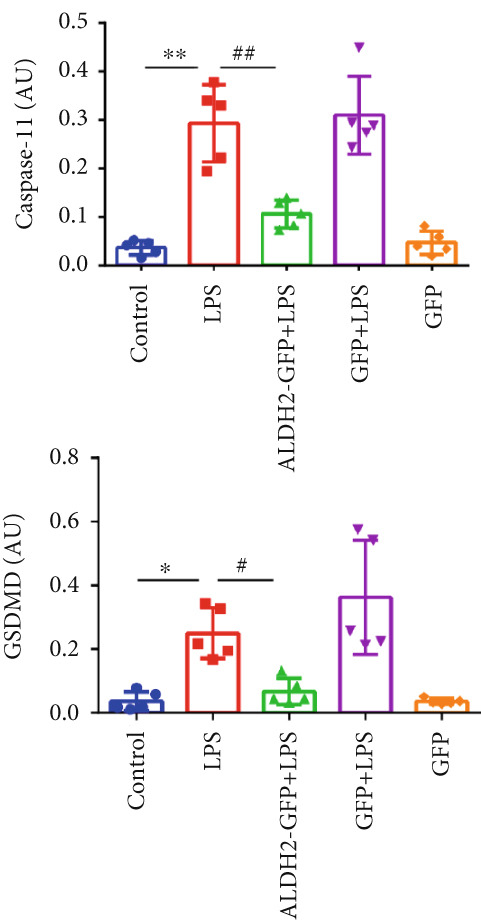
(b)

## 4. Discussion

Severe sepsis or septic shock triggers life‐threatening organ dysfunction, with the fate of individual organs exhibiting marked interdependence: dysfunction or failure of one organ frequently propagates to others, and this cascading effect is particularly prominent in cardiovascular injury [7, 18]. Cardiac dysfunction often develops into septic cardiomyopathy, in which left and/or right ventricular impairment during systole or diastole, inadequate cardiac output and oxygen delivery, primary myocardial cellular injury, acutely depressed LVEF with ventricular dilation, and ultimately involves multiple organ functional injury in the body [8].

The pathogenesis of sepsis is remarkably complex, encompassing a spectrum of cellular and molecular pathophysiological processes—including dysregulated inflammatory responses, immune dysfunction, mitochondrial damage, coagulation disorders, neuroendocrine‐immune network abnormalities, and endoplasmic reticulum stress [8]. Among these, the imbalance of inflammatory responses stands as the most critical driver of sepsis progression, persisting throughout the entire pathological course. In addition to the dysregulation of inflammatory and immune function, various cell death modes (including pyroptosis, necrosis, and apoptosis) are activated during sepsis. While these modes initially contribute to host defense against pathogens, their excessive activation under septic conditions exacerbates tissue injury and organ dysfunction [7]. Among these, noncanonical pyroptosis has been identified as a critical pathway in sepsis pathogenesis, primarily triggered by the sensing of intracellular LPS. Kayagaki et al. [4] indicated that caspase‐11, rather than caspase‐1, is the foremost reason for septic mouse death; caspase‐11 knockdown mice delayed the existence of sepsis. Another research [10, 12] pointed out that sepsis is caused by caspase‐11‐mediated noncanonical pyroptosis, which was elicited by LPS. As highlighted in our study, SIMD exacerbates hemodynamic instability, impairs cardiac output, and further compromises the perfusion of vital organs, creating a vicious cycle of multiorgan dysfunction.

ALDH2 is best known for its role in acetaldehyde metabolism—facilitating the detoxification of acetaldehyde, a toxic byproduct of ethanol metabolism, to acetate. It has been widely recognized that ALDH2 polymorphism influences the development of various health impairments, including cardiovascular disease, diabetes mellitus, cancer, liver disease, and neurodegenerative disorder [19]. For the cardioprotective effects, accumulating evidence confirms that ALDH2 activation mitigates cardiac injury across multiple pathological contexts: acute myocardial infarction, coronary artery disease, and myocardial ischemia–reperfusion injury [20]. Our previous studies [21, 22] also demonstrated that ALDH2 overexpression alleviates high glucose‐induced myocardial injury.

As a widely recognized protective agent, ALDH2 plays a key inhibitory role in pyroptosis by regulating multiple signaling pathways [19]: ALDH2 overexpression inhibits the activation of the NLRP3 inflammasome and reduces the occurrence of pyroptosis by suppressing the production of reactive oxygen species (ROS); failure of ALDH2 activation leads to the accumulation of ROS and the inhibition of BCL2, thereby triggering pyroptosis mediated by caspase‐3–gasdermin‐E; inhibition of ALDH2 promotes the translocation of histone deacetylase 3 (HDAC3) from the nucleus to mitochondria, and HDAC3 in mitochondria promotes the deacetylation of the alpha subunit of hydroxy‐CoA dehydrogenase, thereby activating the ROS‐mitochondrial DNA (mtDNA)–NLRP3 axis and promoting pyroptosis. Previous studies have mainly focused on the role of ALDH2 in canonical pyroptosis, while our study supplements that ALDH2 exerts a protective effect in the noncanonical pyroptosis pathway.

Collectively, our findings not only identify a novel mechanism that ALDH2 alleviates septic myocardial injury (targeting noncanonical pyroptosis via interaction with GSDMD, HMGB1, and RAGE) but also provide the preclinical evidence that targeting ALDH2—via agonists like Alda‐1 or other ALDH2‐upregulating strategies—could serve as a valuable therapeutic approach for sepsis‐induced cardiac injury. This work thus lays a foundation for future translational studies exploring ALDH2 as a potential therapeutic target to improve outcomes in patients with SIMD.

## Conflicts of Interest

The authors declare no conflicts of interest.

## Author Contributions

Conceptualization: Huan Liang, Hongwei Ye, JiaHui Wang, and Qin Gao. Methodology: Huan Liang, Hongwei Ye, JiaHui Wang, and Qin Gao. Validation: Huan Liang, JiaHui Wang, Yuying He, and Yiren Wang. Writing—original draft: Huan Liang, JiaHui Wang, Hongwei Ye, and Qin Gao. Data curation: Huan Liang, JiaHui Wang, Yuying He, and Yiren Wang. Review and editing: Huan Liang, JiaHui Wang, and Qin Gao.

## Funding

The study is supported by the Anhui Province Education Major Project (2022AH040213), the Anhui Province Excellent Scientific Research and Innovation Team Project (2022AH010083), and the Research Project of Bengbu Medical University (2024byfy004, 2024byfy005).

## Data Availability

The data that support the findings of this study are available from the corresponding authors upon reasonable request.
